# Outbreak tracking of Aleutian mink disease virus (AMDV) using partial NS1 gene sequencing

**DOI:** 10.1186/s12985-017-0786-5

**Published:** 2017-06-21

**Authors:** P. Ryt-Hansen, C.K. Hjulsager, E.E. Hagberg, M. Chriél, T. Struve, A.G. Pedersen, L.E. Larsen

**Affiliations:** 1 National Veterinary Institute, Technical University of Denmark, Bülowsvej 27, DK-1870 Frederiksberg C, Denmark; 2 Kopenhagen Fur, Langagervej 60, DK-2600 Glostrup, Denmark; 3 Center for Biological Sequence Analysis, Technical University of Denmark, Kemitorvet Building 208, -2800 Lyngby, DK Denmark; 4 Broparken 27, DK-2680 Solrød Strand, Denmark

**Keywords:** Plasmacytosis, Amdv, Carnivore amdoparvovirus, Aleutian mink disease, Denmark, NS1, Phylogenetic analysis

## Abstract

**Background:**

Aleutian Mink Disease (AMD) is an infectious disease of mink (*Neovison vison)* and globally a major cause of economic losses in mink farming. The disease is caused by Aleutian Mink Disease Virus (AMDV) that belongs to the genus *Amdoparvovirus* within the *Parvoviridae* family. Several strains have been described with varying virulence and the severity of infection also depends on the host’s genotype and immune status. Clinical signs include respiratory distress in kits and unthriftiness and low quality of the pelts. The infection can also be subclinical.

Systematic control of AMDV in Danish mink farms was voluntarily initiated in 1976. Over recent decades the disease was mainly restricted to the very northern part of the country (Northern Jutland), with only sporadic outbreaks outside this region. Most of the viruses from this region have remained very closely related at the nucleotide level for decades. However, in 2015, several outbreaks of AMDV occurred at mink farms throughout Denmark, and the sources of these outbreaks were not known.

**Methods:**

Partial NS1 gene sequencing, phylogenetic analyses data were utilized along with epidemiological to determine the origin of the outbreaks.

**Results:**

The phylogenetic analyses of partial NS1 gene sequences revealed that the outbreaks were caused by two different clusters of viruses that were clearly different from the strains found in Northern Jutland. These clusters had restricted geographical distribution, and the variation within the clusters was remarkably low. The outbreaks on Zealand were epidemiologically linked and a close sequence match was found to two virus sequences from Sweden. The other cluster of outbreaks restricted to Jutland and Funen were linked to three feed producers (FP) but secondary transmissions between farms in the same geographical area could not be excluded.

**Conclusion:**

This study confirmed that partial NS1 sequencing can be used in outbreak tracking to determine major viral clusters of AMDV. Using this method, two new distinct AMDV clusters with low intra-cluster sequence diversity were identified, and epidemiological data helped to reveal possible ways of viral introduction into the affected herds.

**Electronic supplementary material:**

The online version of this article (doi:10.1186/s12985-017-0786-5) contains supplementary material, which is available to authorized users.

## Background

Aleutian Mink Disease Virus (AMDV) is a single stranded DNA virus belonging to the *Amdovirus* genus and the family *Parvoviridae*. The genome of AMDV is approximately 4.8 kilobases long and consists of two structural proteins (VP1 and VP2) and three non-structural proteins (NS1, NS2 and NS3). The NS1 gene is of particular importance as it plays a key role in viral replication, and it shows a high degree of genetic variability between different strains [1, 2, 3].

AMDV is the cause of Aleutian mink disease (AMD) with different disease manifestations in mink. The pups may develop interstitial pneumonia leading to high mortality, whereas the adults often develop chronic disease [4]. This chronic disease is characterized as an immune mediated disease, where the development of immune complexes in different organs lead to an increased mortality rate and lower fertility and thereby affects the mink production significantly [4–6]. Some mink become subclinically infected with only minor impact on the production. These mink may act as carriers of the disease and pose a risk for introducing new infections in the population [4]. The virulence of the different AMDV strains varies from highly virulent types to low virulent types [1, 7]. A reservoir of different AMDV strains persists in wild mink [8–11]. Additionally, AMDV also infect other wild living *mustelidae* e.g. raccoons, weasel, ferrets, otters, skunks and badgers [9, 12].

**Fig. 1:**
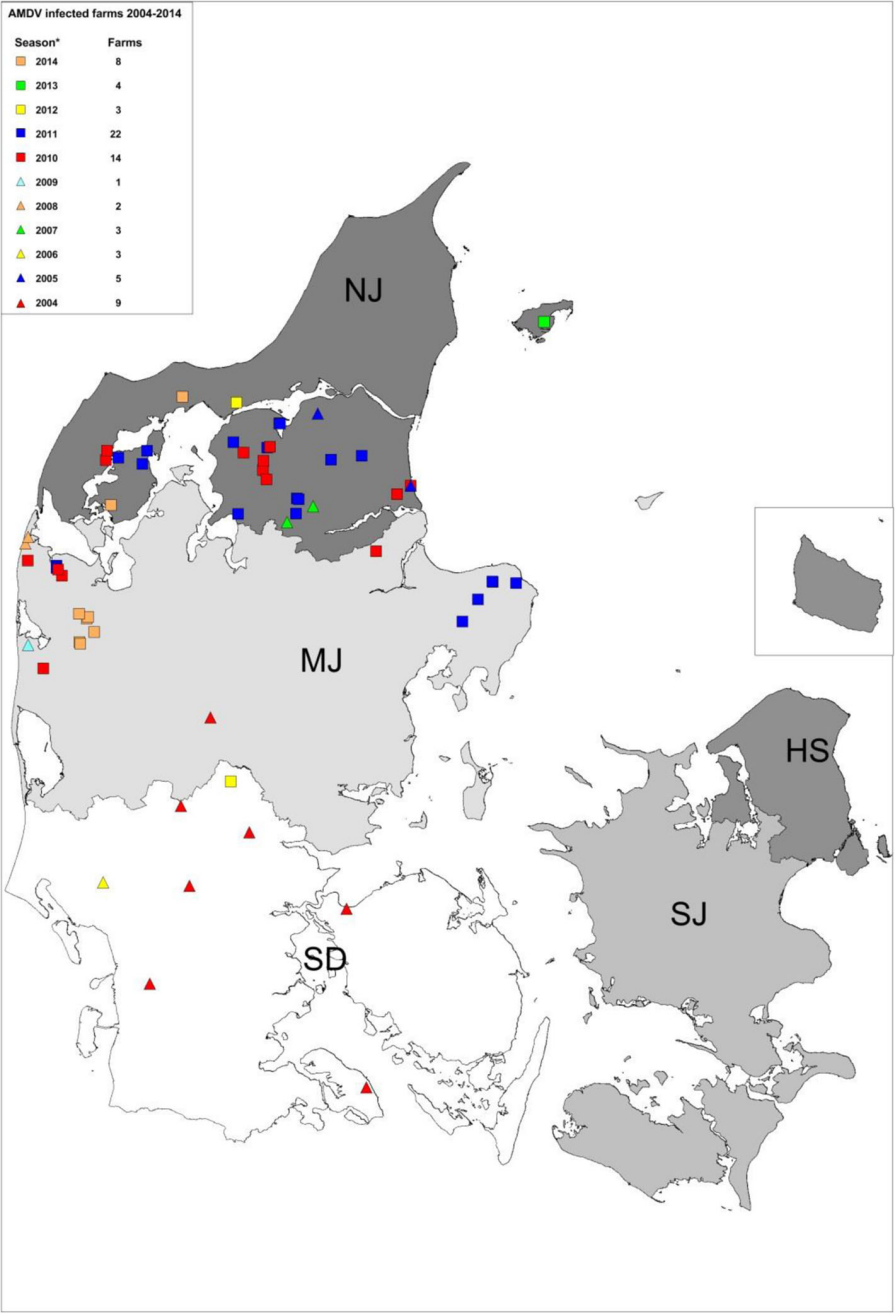
Information of the location and number of sporadic cases of AMDV south of the Northern peninsula of Jutland from 2004 to 2014. NJ (Northern Jutland), MJ (Middle Jutland), SD (Southern Denmark), Zealand (SJ), HS (the regional capital and Bornholm). * from the beginning of April to the end of March

In Denmark, a voluntary test and stamping out policy of AMDV positive mink was initiated in 1976, and supported by legislation in 1999 in order to verify the AMDV status of all Danish mink farms. In this legislation, a farm is defined as infected if three or more mink are tested positive by an AMDV antibody test or if AMDV is detected. If a farm has between 1 and 2 animals testing positive, the farm is subjected to additional tests and no live mink may leave the farm [13]. The herd prevalence of AMDV in Danish farmed mink has been 5 % or lower since 2001 and AMDV positive farms were almost exclusively located in the northern part of Jutland with the exception of a single feed-borne outbreak in the southern part of Jutland in 2002 [14] and few sporadic detections in mainland Jutland over the recent years (Fig. 1). However, in the autumn 2015 and onwards, multiple AMDV positive farms were detected in several locations across Denmark by the routine control program. Previous studies have shown that different viral strains of AMDVs can be distinguished by phylogenetic analysis based on partial NS1 sequencing [10, 14–17] therefore this method was employed for the investigation of an epidemiological link between these outbreaks based on genetic characterization of the AMDV strains from each outbreak by partial sequencing of the NS1 gene and subsequent phylogenetic analysis.

## Methods

### Samples

Spleens, mesenteric lymph nodes and blood-samples from euthanized AMDV-antibody positive mink were collected by Kopenhagen Fur and submitted to The National Veterinary Institute for further processing. In total, 137 mink from 73 different farms were sampled and analysed. The majority of the samples were obtained during the acute outbreaks but also a number of retrospective samples were analysed dating back to 2003. The retrospective samples constituted thirteen samples from five farms. Information on the geographical position (expressed as the postal code which cover the area around one larger town), feed producer (FP), number of antibody-positive animals at the most recent test, and whether antibody positive animals had been detected in the farm for the past three years was recorded for each sampled farm (Table 1 and Table 2).

**Table 1:** Information on the different farms in the Holstebro cluster and the Saeby cluster

Cluster:	No of farms:	Northern region of Jutland:	Middle or southern part of Jutland	Infected in the last 3 years:
Holstebro	54	8	46	7.4%
Saeby	15	13	2	46%

The information includes the total number of farms in each cluster, the region the farm is located in and if the farms have been infected with AMDV before

**Table 2:** Distribution of feed producer (FP) between the farms infected with the three different main clusters

Cluster:	FP-A	FP-B	FP-C	FP-D	FP-E	Total
Holstebro	0	8	7	2	37	54
Saeby	0	14	0	0	1	15
Zealand	5	0	0	0	0	5
Total farms supplied by this FP:	67	327	193	48	238	

Total indicates the total number of farms within each of the three clusters

### DNA extraction, PCR and sequencing

180 mg tissue was homogenised in 1300 μl ATL buffer (QIAGEN, Copenhagen, Denmark) using a 5 mm stainless steel bead (QIAGEN, Copenhagen, Denmark) in a 2 ml microcentrifuge tube and shaken for 3 min. at 30 Hz on Tissuelyzer II (QIAGEN, Copenhagen, Denmark). Then samples were centrifuged at 12.000 G for 3 min and 200 μl of the supernatant was used for the further extraction. For the blood samples, 200 μl of serum was used. 20 μl of Proteinase K was added to the 200 μl tissue homogenate or serum sample and incubated for 30 min at 56 °C. Total DNA was extracted from the samples using the QIAamp®DNA Mini Kit (QIAGEN) with tissue standard protocol version 1 automated on a QIAcube (Qiagen, Hilden, Germany) according to instructions from the supplier. PCR was performed essentially as previously described [18] with few modifications. Each PCR reaction contained 5 μl of extracted DNA, 5 μl PCR gold buffer, 1 μl dNTPs, 5 μl MgCl, 2.5 μl of each 0.5 μM primer (AMDV-F-7-H-PN1 and AMDV-R-7-HPN2), 28.5 μl Nuclease free water and 0.5 μl AmpliTaq Gold enzyme (AmpliTaq Gold Polymerase kit; Thermo Fisher Scientific, Copenhagen, Denmark). The PCR reactions were run on a T3 PCR machine (Biometra, Fredensborg, Denmark) with the following cycling conditions: 94 °C for 10 min, followed by 45 cycles of denaturation at 94 °C for 30 s, annealing at 55 °C for 30 s and elongation at 72 °C for 30 s and a final elongation at 72 °C for 7 min. The primers generated a 328 base pair sequence covering part of the 5′ end of the NS1 gene and constituting 7% of the full genome. An isolate of the AMDV-G strain (Kopenhagen Diagnostics, Kopenhagen Fur, Denmark) was used as a positive control both for the DNA extraction and for the PCR, nuclease free water (QIAGEN) was used for negative control of extraction and PCR. The PCR products were visualized with UV-light on a 2% agarose E-gel (Thermo Fisher Scientific, Copenhagen, Denmark), purified with High Pure PCR Product Purification Kit (Roche, Hvidovre, Denmark) and Sanger sequenced with the PCR primers at LGC Genomics (Berlin, Germany).

### Phylogenetic analysis

The nucleotide sequences were analysed using the program CLC main workbench version 7.5 (www.clcbio.com, QIAGEN, Aarhus, Denmark). For each sample, sequence chromatogram files resulting from the forward and reverse primers were contiged and proof-read manually. The primer binding regions were trimmed off manually to generate 328 bp long consensus sequences read on both strands from each sample. Each sequence was compared to sequences in NCBI GenBank using the function “BLAST at NCBI” (available at https://blast.ncbi.nlm.nih.gov/Blast.cgi?PAGE_TYPE=BlastSearch).

Existing Danish homologous sequences in GenBank NCBI retrieved in February 2016 were aligned at the nucleotide level with consensus sequences of this study, using the “MUSCLE” alignment algorithm. Analysis of nucleotide sequence identities between sequences and clusters was performed with the “pairwise comparison” functionality in CLC based on the alignment.

The best fitting substitution model for this dataset was determined using jModelTest version 2.1.10 [19]. The data were tested against eleven different substitution models using the command “compute likelihood scores”. A Bayesian tree was then generated with software MrBayes version 3.2.6 [20] with the optimal substitution model running for 10.000.000 generations and with samples drawn every 1000 steps. The phylogenetic tree was inferred in a Bayesian framework and with MCMC sampling of posterior probabilities. Tree visualization was performed in FigTree version 1.4 (http://tree.bio.ed.ac.uk/software/figtree/). The sequences were assigned a unique identifier and were named according to their region of origin, sampling date and feed producer, e.g. AMDV_mink-f_DK_NJ_20–1-16_2016–02-15_FP-F. The different geographical regions of Denmark are indicated in Fig. 1.

The different clusters in the tree were assigned different branch colours and each sequence taxon were colour coded according to their feed producer. Further information on the individual sequences and a detailed phylogenetic tree can be found in the Additional file 1.

## Results

### Phylogenetic analysis and sequence analysis:

**Fig. 2:**
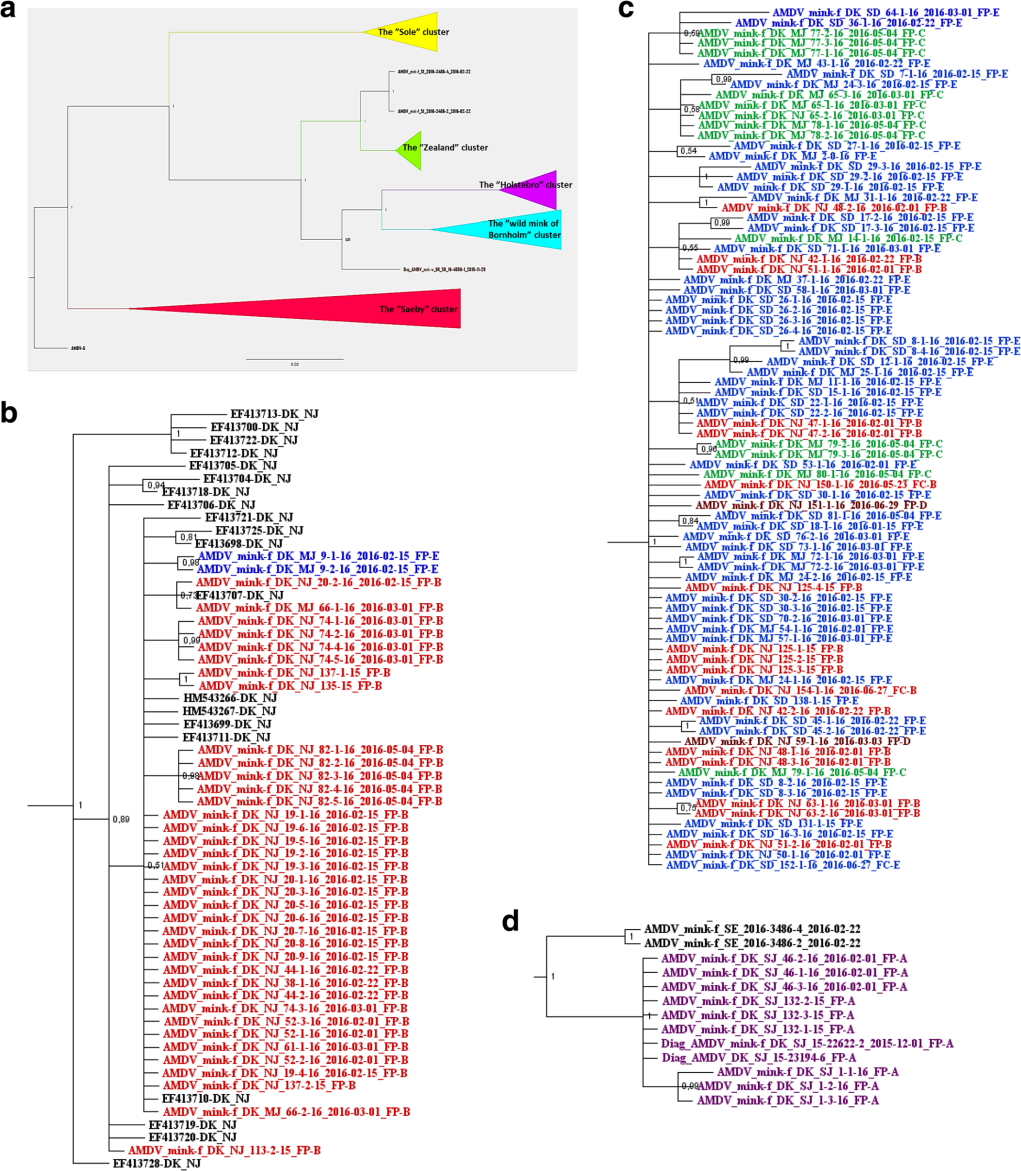
**a** Bayesian tree of the partial NS1 from this study along with other Danish sequences from GenBank. Red cluster: the “Saeby” cluster, purple cluster: the “Holstebro” cluster, green cluster: the “Zealand” cluster, turkois cluster: the “wild mink of Bornholm” cluster and the yellow cluster: the “Sole” cluster. Danish wild mink: Brown taxon. Two Swedish sequences: Black Taxon. **b** Enlargement of the “Saeby” Cluster from Fig. 2a. Feed suppliers are named as follows; FP-A: purple taxon, FP-B: red taxon, FP-C: green taxon, FP-D: brown taxon, FP-E: Blue taxon. **c** Enlargement of the “Holstebro” cluster from Fig. 2a. Feed suppliers are named as follows; FP-A: purple taxon, FP-B: red taxon, FP-C: green taxon, FP-D: brown taxon, FP-E: Blue taxon. **d** Enlargement of the “Zealand” cluster from Fig. 2a: Feed suppliers are named as follows; FP-A: purple taxon, FP-B: red taxon, FP-C: green taxon, FP-D: brown taxon, FP-E: Blue taxon. Swedish sequences: black taxon

The best substitution model for the sequences included in this study was the GTR model with a gamma-distributed rate variation across sites. The Bayesian phylogenetic tree (Fig. 2a) showed that AMDV sequences from the sampled Danish farmed mink felt into three major clusters.

One cluster was closely related to the Saeby strain that has been circulating in the peninsula of Northern Jutland for at least four decades [14] (Fig. 2b). These sequences were all obtained from farms located around the peninsula except for two cases from the middle region of Jutland.

The two other clusters did not closely resemble any known sequences of AMDVs available from Genbank. However, these two clusters corresponded to the geographical location of the farms, with one cluster consisting of sequences isolated only in Jutland and Funen (“the Holstebro cluster”) (Fig. 2c), and the other containing sequences isolated only on Zealand (“the Zealand cluster”) (Fig. 2d).

The sequences obtained from GenBank from previous Danish studies also clustered according to their geographical location. Thus, sequences from wild mink from Bornholm island were found in one cluster, sequences from farms in a feed-borne outbreak related to the town Sole in 2002 in a second, and sequences from farms infected with the Saeby strain in a third cluster (Fig. 2a).

Pairwise comparisons showed that the sequences in the two new Danish clusters (“The Holstebro cluster” and the “Zealand cluster”) had a within cluster sequence identity of 97.3–100%. The Saeby cluster included 2015/2016 sequences as well as sequences from previous studies and the within cluster sequence identity was similar to that of the two other clusters. However, the 2015/2016 Saeby sequences have a sequence identity of 99.3–100%, which corresponds to a difference of at most 2 nucleotide positions. The identities between all the Danish clusters varied between 81.1–94.2% (Table 3).

**Table 3:** Sequence identity between the different Danish clusters seen in Fig. 2a

Clusters compared	Sequence identity %
Sæby – Holstebro	84.15–86.59
Sæby – Zealand	86.59–87.50
Holstebro – Zealand	90.24–92.38
Sæby – Sole	85.37–87.20
Holstebro – Sole	81.10–84.76
Zealand – Sole	84.76–85.67
Sæby – Bornholm	85.37–88.11
Holstebro – Bornholm	89.63–94.21
Zealand – Bornholm	90.24–92.38

Two AMDV sequences from Sweden were found to be 97.87% identical to the viruses sequenced from the outbreak in Zealand (Fig. 2d), corresponding to 7–8 nucleotide differences (unpublished by the authors). Nucleotide differences resulting in an amino acid change between the three main Danish clusters are seen in Table 4. Only three amino acid changes are unique to each of the three strains, and in total 16 out of 109 amino acids varied between the strains. The 3′ end of the partial NS1 gene seemed to be completely conserved along with the middle of the fragment ranging from nt 142–230. In contrast, the region between base 30–142 and the 5′ end of the partial NS1 was highly variable.

**Table 4:** Point mutations that give rise to amino acid changes between the three main Danish clusters

Position in the partial NS1 sequence:	Saeby cluster Nucleotides: Amino acid:	Holstebro cluster Nucleotides: Amino acid:	Zealand cluster Nucleotides: Amino acid:
32–34	CAA: Q	CAC: H	CAA: Q
50–52	TTT: F	TTG/TTA: L	TTG: L
53–55	ATA: I	CTT: L	ATT: I
59–61	AGA: R	AGA: R	AAA: K
62–64	CTA: L	ATA: I	ATA: I
**80–82**	GTT: V	TGT: C	GCT: A
104–106	GAC: D	AAC/GAC: N/D	GAC: D
107–109	ATA: I	CAA: Q	CAA: Q
131–133	GAT: D	GCT/GAT: A/D	GCT: A
140–142	AAG: K	GAT/AAT/AGT: D/N/S	GAT: D
230–232	AAC: N	ACC: T	AAC: N
248–250	ATT: I	CTA/ATA/ATG: L/I/M	ATG: M
278–280	AAT: N	CAT: H	CAT: H
**287–289**	GGT: G	AGT: S	AAT: N
302–304	TTT: F	TAT: Y	TAT: Y
**323–325**	ATT: I	TTA: L	GGT: V
326–328	ATT: I	ATT: I	GTT: V

The three major strains of Denmark are included: the Saeby strain, the Holstebro strain and the Zealand strain. Mutations unique to each single strain are marked with bold data

Nucleotides: A: adenine, G: guanine, C: cytosine and T: thymine

Aminoacids: A: Alanine, C: cysteine, D aspartic acid, F: phenylalanine, G: glycine, H: histidine, I: isoleucine, K: lysine, L: leucine, M: methionine, N: asparagine, P: proline, Q: glutamine, R: arginine, S: serine, T: threonine, V: valine and Y: tyrosine

### Correlation to feed producers:

Five different feed producers supplied feed to all the infected farms of this study. The phylogenetic relatedness of the three strains and the feed producer used by the individual farm is outlined in the phylogenetic tree (Figure 2b-d) and in Table 2.

#### The Zealand cluster:

All farms from Zealand obtain the feed from the same feed producer FP-A.

#### The Holstebro cluster:

The sampled farms infected with the Holstebro strain obtained feed from four different feed producers. The majority (86.5%) of the farms obtained feed from the “Sole Minkfoder A/S” (FP-E). FP-E solely supplied farms infected with the Holstebro strain (*n* = 37) and non-infected farms, with the exception of one farm infected with the Saeby strain (Table 2).

Additionally seven farms infected with the Holstebro strain obtained feed from “Holstebro Fodercentral” (FP-C), which also supplied a number of non-infected farms (Table 2). Furthermore two farms infected with the Holstebro strain obtained feed from “Hvalpsund Minkfodercentral” (FP-D) and one feed producer “Fodercentralen Limfjorden” (FP-B) was both supplying farms infected with the Holstebro strain (*n* = 8) and farms infected with the Saeby strain (*n* = 14) (Table 2).

#### The Saeby cluster:

All sampled farms infected with the Saeby strain obtained feed from FP-B except one farm located in the middle of Jutland (see above) obtaining feed from FP-E

### Previous outbreaks

From Table 1 it is evitable that 46% of the farms infected with the Saeby strain have been infected with AMDV within the three previous years, whereas only 7.4% of the farms infected with the Holstebro strain have been previously infected with AMDV in that period. Furthermore, 51% of the previously infected farms that belong to the Holstebro cluster had their latest infection in the Sole outbreak in 2002 (unpublished data).

Few sporadic outbreaks of AMDV south of the northern peninsula of Jutland have occurred during the last decade (Fig. 1).

All sequences from this study are available in GenBank with the accession numbers: MF073922 - MF074058

## Discussion

This study demonstrated that partial NS1 sequencing can be used to group sequences in major clusters in case of outbreaks of AMDV. Two new strains/clusters of AMDV in Denmark were found in this study.

The phylogenetic analysis confirmed that the partial NS1 gene sequences can be used to distinguish between major clusters, but inadequate to track virus spread within clusters due to the high level of intra-cluster sequence identity. In this study the geographical location of outbreaks was highly correlated to the clustering of the corresponding isolates.

The high level of sequence identity within each cluster was expected since the samples were taken within a very limited period of time. This also indicated that each outbreak cluster originated from a single source. The high sequence identity recorded in the 2015/2016 sequences of the Saeby cluster probably reflect an evolutionary bottleneck, limiting the genetic diversity of this AMDV strain and is in accordance with a previous study of the evolution of the Saeby strain [14].

The sequence diversity among the Danish clusters (ranging from 8 to 19% difference) is unusually high for a DNA virus, but is in accordance with other studies on AMDV [1, 8, 10, 11, 15–17] and other parvoviruses [21, 22].

A distinct cluster was detected on Zealand where feed was supplied by a single producer. Interestingly, information from farmers revealed that these five farms on Zealand were epidemiologically linked through sharing facilities and staff and therefore the feed producer was not regarded as the source of transmission. The origin of the virus remains unknown, but ongoing studies of the phylogenetic pattern of the NS1 gene from mink in other countries has revealed that the strain found in the outbreak of Zealand resembles Swedish sequences. The sequence identity between the Zealand strains and the Swedish strain was up to 97.9% equal to a nucleotide difference of seven, which is the same identity as seen within each of the Danish clusters, suggesting a possible link. AMDV has been shown to be very resistant and capable of persisting in the environment and clothing, and can therefore easily be transmitted between farms [23–26].

The second cluster, the Holstebro cluster, was only seen in Jutland and Funen with the majority of the farms being located in the southern and middle region of Jutland. In addition to a clear geographical pattern of the sequences within this cluster, a possible connection to the feed producer was suspected. In total four feed producers were supplying the farms infected with the new Holstebro strain. Two of these feed producers (FP-C and FP-E) were supplying feed to the majority of the farms infected with the Holstebro strain along with non-infected farms. Only one exception of FP-E supplying a farm infected with the Saeby strain was discovered and this case will be discussed separately. Neighbouring farms to the farms infected with the Holstebro strain with another feed producer were not infected, which implied a lack of horizontal transmission from neighbours, which in turn suggested the feed as the most likely route of transmission. If the feed was contaminated with AMDV it is most likely that the virus would be in-homogenously distributed in the feed and therefore it is unlikely that all farms obtaining feed from the involved feed supplier would have been exposed to infectious doses of the virus. This could explain why only some of the farms obtaining feed from a specific feed producer were infected. The fact that the sequences within the Holstebro cluster were so homogeneous suggested that the feed producers has either all bought the same feed ingredient contaminated with the same source of the virus, or that the different feed producers have bought different feed ingredients contaminated with the same source of the virus. The likelihood of persistence of infectious AMDV in mink feed for a long time is high and previous studies have confirmed that AMDV is very difficult to inactivate [24–26]. Unfortunately it was not possible to test the feed for presence of AMDV, since feed batches are delivered to farms and eaten the same day.

During a major feed borne outbreak in 2002, similar homogeneity between the strains was observed. All infected farms in this outbreak were supplied with feed from the Sole feed producer and 223 out of 310 farms were infected with AMDV. Additional 44 farms in the neighbouring area of the farms, but supplied by a different feed producer, were also sampled and only one of these farms was found positive [27].

One feed producer (FP-B) delivered feed to eight farms infected with the Holstebro strain, but also to the majority of the farms already infected with the Saeby strain. Therefore, it cannot be ruled out that some of these eight farms acquired a double infection even though only the Saeby strain was detected in these herds. At the time of sampling, the number of antibody positive animals in the Saeby strain infected farms seemed to be higher than in farms infected with the Holstebro strain (unpublished data).Thus, if double infected herds occurred the chance of sampling a Saeby infected mink were higher than for sampling a mink infected with the Holstebro strain. Nevertheless, a number of farms supplied by FP-B were found negative for AMDV indicating that not all herds receiving the feed from FP-B became infected, maybe because they were not exposed to an infectious dose of the Holstebro strain in the feed. The epidemiological information of the eight farms infected with the Holstebro strain obtaining feed from FP-B, revealed that these farms were all located near the border of the peninsula of northern Jutland, and also in neighbouring cities. In contrast, the majority of the farms infected with the Saeby strain obtaining feed from FP-B were located in the very north of the peninsula. Thus another explanation of these farms being infected with the Holstebro strain could be horizontal transmission between farms. Additionally, there were two farms infected with the Holstebro strain, which obtained feed from FP-D. Other farms obtaining feed from this feed producer were not infected, and one of the two farms was located in the same area of the eight above mentioned farms. This may again indicate horizontal transmission of AMDV between farms.

The two farms infected with the Saeby strain, which is geographically located in the middle of Jutland, seemed to be special cases of the Saeby strain having spread outside of Northern peninsula of Jutland by horizontal transmission. The probability of these cases having a correlation to the feed is very low as outbreaks of the Saeby strain south of the peninsula have either not been recorded or only been detected as single sporadic cases. As shown in Fig. 1, there have been recurrent sporadic cases of AMDV south of the peninsula, which demonstrated that secondary transmission of the virus is unavoidable and a constant threat to the regions free of the virus. Another potential source for AMDV outbreaks could be spread from wild living mustelids. Several mustelid species have been tested positive for AMDV [8, 9, 13]. During the sampling period of the present study, only one wild mink were found antibody positive in the routine diagnostic surveillance program at the National Veterinary Institute, Technical University of Denmark and the partial NS1 sequence obtained was very different from any of the Danish clusters (Fig. 2a). Thus, it is unlikely that the AMDV from this wild mink was the source of any of the outbreaks, but test of more wild living mink should be performed before it can be excluded that wild living mustelids act as a reservoir for AMDV in Denmark.

Within cluster analysis to determine the route of transmission between farms would require more sequence information. The limitation of the interpretation of the partial NS1 data could be avoided in future studies by using next generation sequencing (NGS) of the full genome. This method would allow for trace of the transmission of the virus between farms and to draw more valid conclusion using the full genome sequencing compared to the partial NS1 [28]. Furthermore, NGS would ease the identification of herds infected with more than one strain as previously reported [29].

The test and stamp out strategy, disinfection of the farm and limiting of possible transmission routes between infected farms is crucial [23]. The last outbreak in 2002 with the Sole strain was controlled this way and no further outbreaks with this strain have been recorded since 2004. If a farm become infected it is possible via thorough biosecurity measures to minimize the risk for spread of the virus to the neighbouring farms and within the farm.

## Conclusion

This study confirmed that partial NS1 sequencing can be used to determine major viral clusters in case of outbreaks of AMDV and identified two new distinct AMDV clusters with low intra-cluster sequence diversity.

An epidemiological link between the outbreaks on Zealand was potentiated and it was shown that the Zealandic strains clustered and resembled Swedish strains. The Holstebro outbreak on the other hand was more complex and the high homogeneity within the Holstebro cluster suggests either one introduction of virus of the same origin or several introductions with the same source. Outbreaks caused by contaminated feed were documented for two feed producers (FP-C and FP-E) and possibly also from a third (FP-B).

## Additional file

Additional file 1: Supplementary material_phylogenetic tree. (PDF 1433 kb)

## Abbreviations

AMDV: Aleutian mink disease virus
Bp/bps: Base pair/base pairs
CIEP: Counter immunoelectrophoresis
NGS: Next generation sequencing
NS1: Nonstructural protein 1
Nt: nucleotide
PCR: Polymerase chain reaction
