# Supplementary material for: Outbreak tracking of Aleutian mink disease virus (AMDV) using partial NS1 gene sequencing
Source: Virol J. 2017 Jun 21;14:119. doi: 10.1186/s12985-017-0786-5 (PMC5480136; doi:10.1186/s12985-017-0786-5)
Supplement: Supplementary file 1 — Supplementary material_phylogenetic tree. (PDF 1433 kb) [file 12985_2017_786_MOESM1_ESM.pdf]

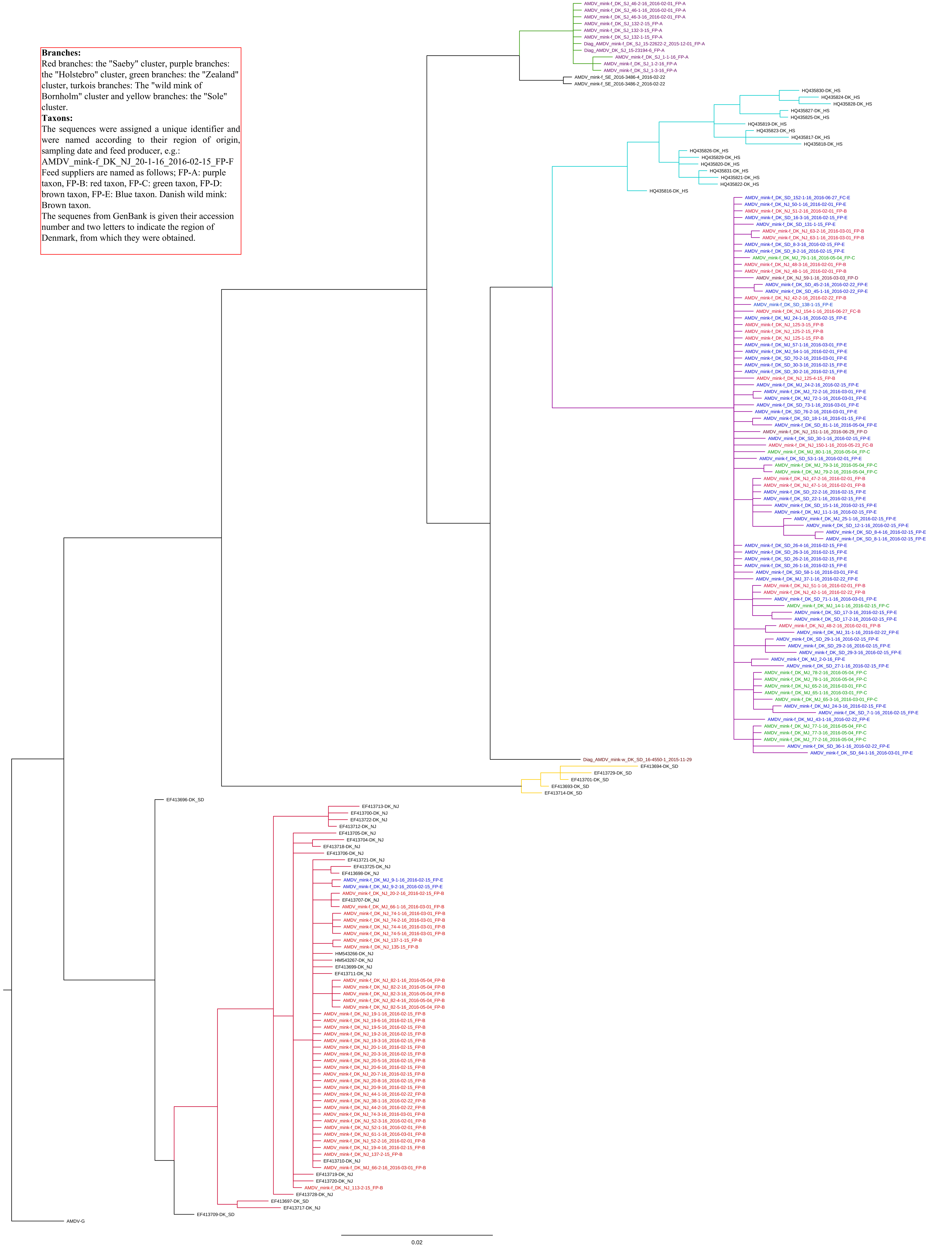

Branches:
Red branches: the "Saeby" cluster, purple branches: the "Holstebro" cluster, green branches: the "Zealand" cluster, turkois branches: The "wild mink of Bornholm" cluster and yellow branches: the "Sole" cluster.
Taxons:
The sequences were assigned a unique identifier and were named according to their region of origin, sampling date and feed producer, e.g.: AMDV_mink-f_DK_NJ_20-1-16_2016-02-15_FP-F
Feed suppliers are named as follows; FP-A: purple taxon, FP-B: red taxon, FP-C: green taxon, FP-D: brown taxon, FP-E: Blue taxon. Danish wild mink: Brown taxon.
The sequenes from GenBank is given their accession number and two letters to indicate the region of Denmark, from which they were obtained.
AMDV_mink-f_DK_SJ_46-2-16_2016-02-01_FP-A
AMDV_mink-f_DK_SJ_46-1-16_2016-02-01_FP-A
AMDV_mink-f_DK_SJ_46-3-16_2016-02-01_FP-A
AMDV_mink-f_DK_SJ_132-2-15_FP-A
AMDV_mink-f_DK_SJ_132-3-15_FP-A
AMDV_mink-f_DK_SJ_132-1-15_FP-A
Diag_AMDV_mink-f_DK_SJ_15-22622-2_2015-12-01_FP-A
Diag_AMDV_DK_SJ_15-23194-6_FP-A
AMDV_mink-f_DK_SJ_1-1-16_FP-A
AMDV_mink-f_DK_SJ_1-2-16_FP-A
AMDV_mink-f_DK_SJ_1-3-16_FP-A
AMDV_mink-f_SE_2016-3486-4_2016-02-22
AMDV_mink-f_SE_2016-3486-2_2016-02-22
HQ435830-DK_HS
HQ435824-DK_HS
HQ435828-DK_HS
HQ435827-DK_HS
HQ435825-DK_HS
HQ435819-DK_HS
HQ435823-DK_HS
HQ435817-DK_HS
HQ435818-DK_HS
HQ435826-DK_HS
HQ435829-DK_HS
HQ435820-DK_HS
HQ435831-DK_HS
HQ435821-DK_HS
HQ435822-DK_HS
HQ435816-DK_HS
AMDV_mink-f_DK_SD_152-1-16_2016-06-27_FC-E
AMDV_mink-f_DK_NJ_50-1-16_2016-02-01_FP-E
AMDV_mink-f_DK_NJ_51-2-16_2016-02-01_FP-B
AMDV_mink-f_DK_SD_16-3-16_2016-02-15_FP-E
AMDV_mink-f_DK_SD_131-1-15_FP-E
AMDV_mink-f_DK_NJ_63-2-16_2016-03-01_FP-B
AMDV_mink-f_DK_NJ_63-1-16_2016-03-01_FP-B
AMDV_mink-f_DK_SD_8-3-16_2016-02-15_FP-E
AMDV_mink-f_DK_SD_8-2-16_2016-02-15_FP-E
AMDV_mink-f_DK_MJ_79-1-16_2016-05-04_FP-C
AMDV_mink-f_DK_NJ_48-3-16_2016-02-01_FP-B
AMDV_mink-f_DK_NJ_48-1-16_2016-02-01_FP-B
AMDV_mink-f_DK_NJ_59-1-16_2016-03-03_FP-D
AMDV_mink-f_DK_SD_45-2-16_2016-02-22_FP-E
AMDV_mink-f_DK_SD_45-1-16_2016-02-22_FP-E
AMDV_mink-f_DK_NJ_42-2-16_2016-02-22_FP-B
AMDV_mink-f_DK_SD_138-1-15_FP-E
AMDV_mink-f_DK_NJ_154-1-16_2016-06-27_FC-B
AMDV_mink-f_DK_MJ_24-1-16_2016-02-15_FP-E
AMDV_mink-f_DK_NJ_125-3-15_FP-B
AMDV_mink-f_DK_NJ_125-2-15_FP-B
AMDV_mink-f_DK_NJ_125-1-15_FP-B
AMDV_mink-f_DK_MJ_57-1-16_2016-03-01_FP-E
AMDV_mink-f_DK_MJ_54-1-16_2016-02-01_FP-E
AMDV_mink-f_DK_SD_70-2-16_2016-03-01_FP-E
AMDV_mink-f_DK_SD_30-3-16_2016-02-15_FP-E
AMDV_mink-f_DK_SD_30-2-16_2016-02-15_FP-E
AMDV_mink-f_DK_NJ_125-4-15_FP-B
AMDV_mink-f_DK_MJ_24-2-16_2016-02-15_FP-E
AMDV_mink-f_DK_MJ_72-2-16_2016-03-01_FP-E
AMDV_mink-f_DK_MJ_72-1-16_2016-03-01_FP-E
AMDV_mink-f_DK_SD_73-1-16_2016-03-01_FP-E
AMDV_mink-f_DK_SD_76-2-16_2016-03-01_FP-E
AMDV_mink-f_DK_SD_18-1-16_2016-01-15_FP-E
AMDV_mink-f_DK_SD_81-1-16_2016-05-04_FP-E
AMDV_mink-f_DK_NJ_151-1-16_2016-06-29_FP-D
AMDV_mink-f_DK_SD_30-1-16_2016-02-15_FP-E
AMDV_mink-f_DK_NJ_150-1-16_2016-05-23_FC-B
AMDV_mink-f_DK_MJ_80-1-16_2016-05-04_FP-C
AMDV_mink-f_DK_SD_53-1-16_2016-02-01_FP-E
AMDV_mink-f_DK_MJ_79-3-16_2016-05-04_FP-C
AMDV_mink-f_DK_MJ_79-2-16_2016-05-04_FP-C
AMDV_mink-f_DK_NJ_47-2-16_2016-02-01_FP-B
AMDV_mink-f_DK_NJ_47-1-16_2016-02-01_FP-B
AMDV_mink-f_DK_SD_22-2-16_2016-02-15_FP-E
AMDV_mink-f_DK_SD_22-1-16_2016-02-15_FP-E
AMDV_mink-f_DK_SD_15-1-16_2016-02-15_FP-E
AMDV_mink-f_DK_MJ_11-1-16_2016-02-15_FP-E
AMDV_mink-f_DK_MJ_25-1-16_2016-02-15_FP-E
AMDV_mink-f_DK_SD_12-1-16_2016-02-15_FP-E
AMDV_mink-f_DK_SD_8-4-16_2016-02-15_FP-E
AMDV_mink-f_DK_SD_8-1-16_2016-02-15_FP-E
AMDV_mink-f_DK_SD_26-4-16_2016-02-15_FP-E
AMDV_mink-f_DK_SD_26-3-16_2016-02-15_FP-E
AMDV_mink-f_DK_SD_26-2-16_2016-02-15_FP-E
AMDV_mink-f_DK_SD_26-1-16_2016-02-15_FP-E
AMDV_mink-f_DK_SD_58-1-16_2016-03-01_FP-E
AMDV_mink-f_DK_MJ_37-1-16_2016-02-22_FP-E
AMDV_mink-f_DK_NJ_51-1-16_2016-02-01_FP-B
AMDV_mink-f_DK_NJ_42-1-16_2016-02-22_FP-B
AMDV_mink-f_DK_SD_71-1-16_2016-03-01_FP-E
AMDV_mink-f_DK_MJ_14-1-16_2016-02-15_FP-C
AMDV_mink-f_DK_SD_17-3-16_2016-02-15_FP-E
AMDV_mink-f_DK_SD_17-2-16_2016-02-15_FP-E
AMDV_mink-f_DK_NJ_48-2-16_2016-02-01_FP-B
AMDV_mink-f_DK_MJ_31-1-16_2016-02-22_FP-E
AMDV_mink-f_DK_SD_29-1-16_2016-02-15_FP-E
AMDV_mink-f_DK_SD_29-2-16_2016-02-15_FP-E
AMDV_mink-f_DK_SD_29-3-16_2016-02-15_FP-E
AMDV_mink-f_DK_MJ_2-0-16_FP-E
AMDV_mink-f_DK_SD_27-1-16_2016-02-15_FP-E
AMDV_mink-f_DK_MJ_78-2-16_2016-05-04_FP-C
AMDV_mink-f_DK_MJ_78-1-16_2016-05-04_FP-C
AMDV_mink-f_DK_NJ_65-2-16_2016-03-01_FP-C
AMDV_mink-f_DK_MJ_65-1-16_2016-03-01_FP-C
AMDV_mink-f_DK_MJ_65-3-16_2016-03-01_FP-C
AMDV_mink-f_DK_MJ_24-3-16_2016-02-15_FP-E
AMDV_mink-f_DK_SD_7-1-16_2016-02-15_FP-E
AMDV_mink-f_DK_MJ_43-1-16_2016-02-22_FP-E
AMDV_mink-f_DK_MJ_77-1-16_2016-05-04_FP-C
AMDV_mink-f_DK_MJ_77-3-16_2016-05-04_FP-C
AMDV_mink-f_DK_MJ_77-2-16_2016-05-04_FP-C
AMDV_mink-f_DK_SD_36-1-16_2016-02-22_FP-E
AMDV_mink-f_DK_SD_64-1-16_2016-03-01_FP-E
Diag_AMDV_mink-w_DK_SD_16-4550-1_2015-11-29
EF413694-DK_SD
EF413729-DK_SD
EF413701-DK_SD
EF413693-DK_SD
EF413714-DK_SD
EF413696-DK_SD
EF413713-DK_NJ
EF413700-DK_NJ
EF413722-DK_NJ
EF413712-DK_NJ
EF413705-DK_NJ
EF413704-DK_NJ
EF413718-DK_NJ
EF413706-DK_NJ
EF413721-DK_NJ
EF413725-DK_NJ
EF413698-DK_NJ
AMDV_mink-f_DK_MJ_9-1-16_2016-02-15_FP-E
AMDV_mink-f_DK_MJ_9-2-16_2016-02-15_FP-E
AMDV_mink-f_DK_NJ_20-2-16_2016-02-15_FP-B
EF413707-DK_NJ
AMDV_mink-f_DK_MJ_66-1-16_2016-03-01_FP-B
AMDV_mink-f_DK_NJ_74-1-16_2016-03-01_FP-B
AMDV_mink-f_DK_NJ_74-2-16_2016-03-01_FP-B
AMDV_mink-f_DK_NJ_74-4-16_2016-03-01_FP-B
AMDV_mink-f_DK_NJ_74-5-16_2016-03-01_FP-B
AMDV_mink-f_DK_NJ_137-1-15_FP-B
AMDV_mink-f_DK_NJ_135-15_FP-B
HM543266-DK_NJ
HM543267-DK_NJ
EF413699-DK_NJ
EF413711-DK_NJ
AMDV_mink-f_DK_NJ_82-1-16_2016-05-04_FP-B
AMDV_mink-f_DK_NJ_82-2-16_2016-05-04_FP-B
AMDV_mink-f_DK_NJ_82-3-16_2016-05-04_FP-B
AMDV_mink-f_DK_NJ_82-4-16_2016-05-04_FP-B
AMDV_mink-f_DK_NJ_82-5-16_2016-05-04_FP-B
AMDV_mink-f_DK_NJ_19-1-16_2016-02-15_FP-B
AMDV_mink-f_DK_NJ_19-6-16_2016-02-15_FP-B
AMDV_mink-f_DK_NJ_19-5-16_2016-02-15_FP-B
AMDV_mink-f_DK_NJ_19-2-16_2016-02-15_FP-B
AMDV_mink-f_DK_NJ_19-3-16_2016-02-15_FP-B
AMDV_mink-f_DK_NJ_20-1-16_2016-02-15_FP-B
AMDV_mink-f_DK_NJ_20-3-16_2016-02-15_FP-B
AMDV_mink-f_DK_NJ_20-5-16_2016-02-15_FP-B
AMDV_mink-f_DK_NJ_20-6-16_2016-02-15_FP-B
AMDV_mink-f_DK_NJ_20-7-16_2016-02-15_FP-B
AMDV_mink-f_DK_NJ_20-8-16_2016-02-15_FP-B
AMDV_mink-f_DK_NJ_20-9-16_2016-02-15_FP-B
AMDV_mink-f_DK_NJ_44-1-16_2016-02-22_FP-B
AMDV_mink-f_DK_NJ_38-1-16_2016-02-22_FP-B
AMDV_mink-f_DK_NJ_44-2-16_2016-02-22_FP-B
AMDV_mink-f_DK_NJ_74-3-16_2016-03-01_FP-B
AMDV_mink-f_DK_NJ_52-3-16_2016-02-01_FP-B
AMDV_mink-f_DK_NJ_52-1-16_2016-02-01_FP-B
AMDV_mink-f_DK_NJ_61-1-16_2016-03-01_FP-B
AMDV_mink-f_DK_NJ_52-2-16_2016-02-01_FP-B
AMDV_mink-f_DK_NJ_19-4-16_2016-02-15_FP-B
AMDV_mink-f_DK_NJ_137-2-15_FP-B
EF413710-DK_NJ
AMDV_mink-f_DK_MJ_66-2-16_2016-03-01_FP-B
EF413719-DK_NJ
EF413720-DK_NJ
AMDV_mink-f_DK_NJ_113-2-15_FP-B
EF413728-DK_NJ
EF413697-DK_SD
EF413717-DK_NJ
EF413709-DK_SD
AMDV-G
0.02
